# Major Histocompatibility Complex Class I Chain-Related A (MICA) Molecules: Relevance in Solid Organ Transplantation

**DOI:** 10.3389/fimmu.2017.00182

**Published:** 2017-02-28

**Authors:** Ajay Kumar Baranwal, Narinder K. Mehra

**Affiliations:** ^1^All India Institute of Medical Sciences, New Delhi, Delhi, India

**Keywords:** MICA antibodies, soluble MICA, MICA-129 dimorphism, solid organ transplantation, graft rejection

## Abstract

An ever growing number of reports on graft rejection and/or failure even with good HLA matches have highlighted an important role of non-HLA antigens in influencing allograft immunity. The list of non-HLA antigens that have been implicated in graft rejection in different types of organ transplantation has already grown long. Of these, the Major Histocompatibility Complex class I chain-related molecule A (MICA) is one of the most polymorphic and extensively studied non-HLA antigenic targets especially in the kidney transplantation. Humoral response to MICA antigens has repeatedly been associated with lower graft survival and an increased risk of acute and chronic rejection following kidney and liver transplantation with few studies showing conflicting results. Although there are clear indications of MICA antibodies being associated with adverse graft outcome, a definitive consensus on this relationship has not been arrived yet. Furthermore, only a few studies have dealt with the impact of MICA donor-specific antibodies as compared to those that are not donor specific on graft outcome. In addition to the membrane bound form, a soluble isoform of MICA (sMICA), which has the potential to engage the natural killer cell-activating receptor NKG2D resulting in endocytosis and degradation of receptor–ligand interaction complex leading to suppression of NKG2D-mediated host innate immunity, has been a subject of intense discussion. Most studies on sMICA have been directed toward understanding their influence on tumor growth, with limited literature focusing its role in transplant biology. Furthermore, a unique dimorphism (methionine to valine) at position 129 in the α2 domain categorizes MICA alleles into strong (MICA-129 met) and weak (MICA-129 val) binders of NKG2D receptor depending on whether they have methionine or valine at this position. Although the implications of MICA 129 dimorphism have been highlighted in hematopoietic stem cell transplantation, its role in solid organ transplantation is yet to be explored. This review summarizes the currently available information on MICA antibodies, soluble MICA, and MICA-129 dimorphism in a setting of solid organ transplantation.

## Introduction

The Major Histocompatibility Complex (MHC) class I-related chain genes A and B (MICA and MICB) are a new family of proteins encoded within the human HLA class I genes, first described in 1994 by two independent groups of researchers (1, 2). While the latter group referred to them as Perth beta block transcript 11, Bahram and coworkers named them as MIC, a terminology that was later adopted by the World Health Organization nomenclature committee for factors of the HLA system. Unlike the classical HLA molecules, these proteins are not involved in antigen presentation to T cells. Instead they act as ligands for the activating C-type lectin-like receptor, referred to as natural killer (NK) group 2, member D (NKG2D) which is expressed on NK cells, γδ T cells, and CD8+ αβ T cells. Interaction of MICA with NKG2D leads to activation of antigen-specific cytotoxic T-lymphocyte-mediated cytotoxicity, NK cell responses, and cytokine production (3). Besides, polymorphic MICA antigens are capable of inducing antibodies that may kill target cells in the presence of complement (4). Hence MICA is unique to the extent that it plays a key role in linking the innate and adaptive immune responses in organ transplantation.

## Genetic Aspects and Biochemical Structure

**Figure 1:**
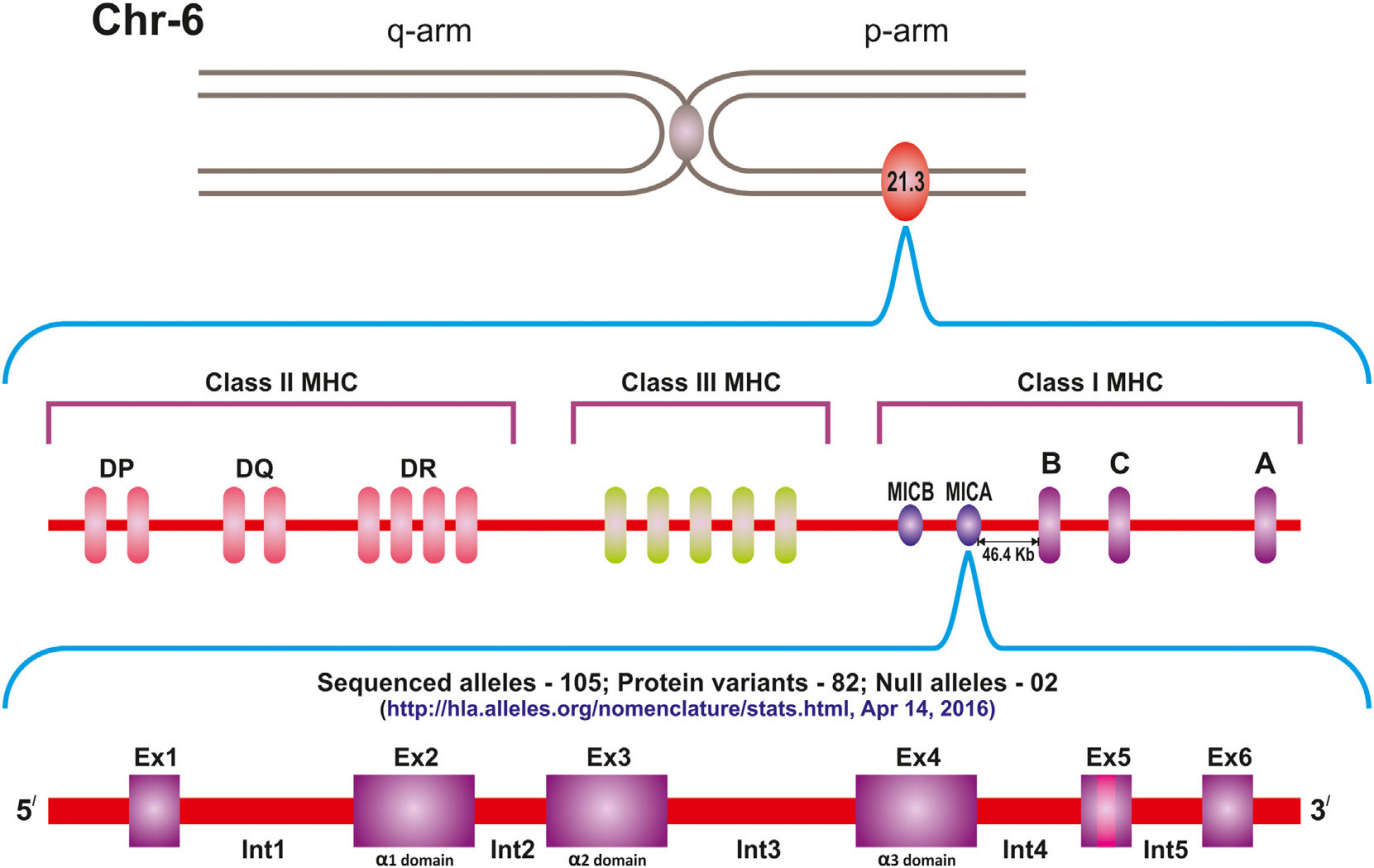
**Location of the MICA gene on the short arm of chromosome 6, centromeric to HLA-B locus**. Currently 105 sequenced alleles and 82 protein variants of the gene are known.

MIC genes are located within the MHC class I region of chromosome 6 p21.3. A total of seven genes, designated as MICA to MICG, have so far been described, of which MICA and MICB are the only functional genes, while MICC to MICG are essentially pseudogenes (5, 6). MICA gene is located centromeric to HLA-B locus at a distance of 46.4 kb, and this close proximity results in a very strong linkage disequilibrium effect between the two (Figure 1).

The domain structure of MICA is much like that of the classical HLA class I molecules with 30% sequence homology and three extracellular domains. Of these, the α1 domain is encoded by exon 2, α2 by exon 3, and α3 by exon 4. The transmembrane (TM) region is encoded by exon 5, while the carboxy-terminal cytoplasmic tail is encoded by exon 6. There are five introns of which the first is the largest intron (7). The gene spans 11.7 kb region and is transcribed into an mRNA of 1,382 bp, which gives rise to 383-amino acid polypeptides of 43 kDa including the leader peptide.

**Figure 2:**
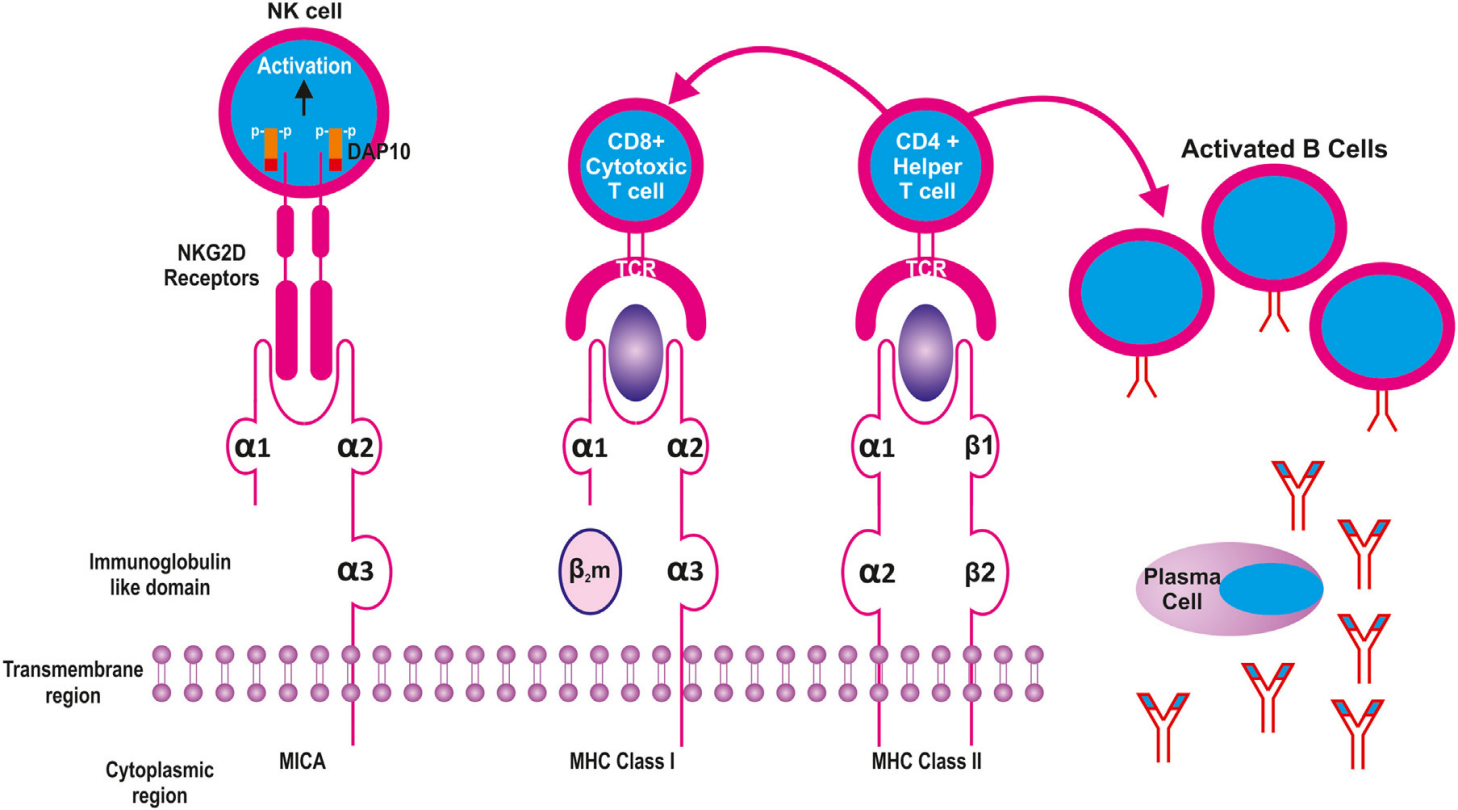
**Structural similarities between Major Histocompatibility Complex (MHC) class I and II molecules with MICA**. The latter is equivalent to the heavy chain of MHC class I molecule without the β2 microglobulin. While the MHC I and II present peptides to CD8 and CD4 cells, respectively, the MICA recognizes NKG2D receptors on the surface of natural killer (NK) cells.

Unlike the HLA class I molecules, the MICA does not bind β_2_-microglobulin (β_2_-m) (Figure 2). Though the structure of MICA looks very similar to its classical class I counterpart, its α-2 helix which is one of the groove defining helices, is disordered and flexible making it unsuitable for peptide binding. Furthermore, as opposed to the HLA class I molecules, the platform formed by the α1 and α2 regions of the MICA molecule points downwards toward the cell membrane thus exposing its underside to the intercellular space. However, when MICA interacts with its receptor NKG2D, the flexible α2 helix becomes ordered by a further two alpha-helical turn and the α1 and α2 domains flip back 96° (8).

## Expression Profile of MIC Proteins

Unlike the ubiquitous expression of classical HLA class I molecules, MIC proteins have limited tissue distribution being expressed constitutively on epithelial cells especially in the gastrointestinal tract (9), endothelial cells, fibroblasts, monocytes, keratinocytes (10), and dendritic cells (11). Zwirner and colleagues demonstrated that MIC molecules are not expressed on resting T or B lymphocytes, and unlike the HLA class I antigens, are not upregulated by INF-γ. Nevertheless, the expression of MICA can be induced on activated CD4+ T cells through release of IL-2 that powerfully induces MICA through calcineurin and other pathways in cooperation with CD3 engagement. Using confocal microscopy, these investigators found low levels of MICA expression on the surface of activated CD4 T cells and stated that this might indicate a protective mechanism of T-cell-dependent NK cell attack (12). MICA through engagement of NK cells helps to accomplish the removal of activated T-cells once the final phase of immune response is completed.

In a study involving total body tissue scan of both MICA and MICB transcription using Northern blot assay, Schrambach et al. reported that both the genes are transcribed in virtually all body tissues except the central nervous system (13). The surface expression of MICA is enhanced under stress conditions such as autoimmune diseases (14), DNA damage (15), ischemia-reperfusion injury (16), viral infections (17), and inflammation (18). Since MICA antigens are also frequently found on tumor cells (19), it implies that they are cell stress markers and their tissular expression is a signal for destruction by NK cells.

## MICA Polymorphism

MICA is the most polymorphic non-classical class I gene known so far with 105 alleles having already been reported and new alleles being continuously identified.^1^ This polymorphism differs from that of the HLA genes in various aspects. First, the magnitude of polymorphism is far less than that seen in the HLA system. Second, in contrast to the HLA class I molecules, where the polymorphism is located predominently in the proximity of antigen binding groove, the MICA polymorphism is dispersed to all the three extracellular domains with the greatest variability in the α2 domain, encoded by exon 3. Another interesting aspect of the polymorphism of MICA is the observed variations in the TM region for several MICA alleles despite having identical extracellular domains. Therefore, it is essential to study polymorphism in the TM region to avoid typing ambiguities (20). Moreover, unlike the polymorphic positions of HLA that typically consists of several amino acids, MICA polymorphism is generated mainly by single amino acid substitutions (except positions 90 and 91) resulting in dimorphism (except residues 156 and 251).

In contrast to MICA, the genetic polymorphism of MICB is limited with a total of 45 alleles reported so far.^2^ There is no concrete evidence to indicate its relevance in transplant outcome.

## MICA-129 Dimorphism

Despite the highly polymorphic nature of MICA genes, only one functional site has been identified that appears to affect the binding of MICA ligands to its receptor NKG2D. Accordingly, a non-synonymous Methionine to Valine change at position 129 of the α2 domain categorizes MICA alleles into “MICA-129 met,” which is a strong binder of NKG2D receptor and “MICA-129 val” having weak binding ability. This dimorphism is identified on a single SNP rs1051792 A>G polymorphism at position 454 in exon 3 of MICA gene, corresponding to amino acid position 129 of the MICA protein. It has been shown that MICA-129 met has a 10- to 50-fold greater capacity to complex NKG2D than those with MICA-129 val (21). The functional consequence of this dimorphism has recently been studied in great details by the group led by Ralf Dressel in Germany (22) who demonstrated that MICA-129 met isoforms are able to induce stronger and faster NKG2D signaling leading to higher degree of NK cell-mediated cytotoxicity and release of INF-γ. This variant was also found to mediate faster co-stimulation and activation of CD8+ T cells. However, such effects were not sustained because the MICA-129 variant was able to induce rapid downregulation of the NKG2D receptor (22). Furthermore, the same group of investigators showed that MICA-129 met isoform is less efficiently expressed on the cell surface as compared to the MICA-129 val variant. This could be due to the intracellular retention of the former and its increased shedding from the cell surface (23). Similarly, like their NKG2D receptor counterparts that according to the polymorphism in the NKC region can be categorized into high NK cell cytotoxicity and low overall cytotoxicity, MICA-129 variants can also associate differently in pathological conditions requiring NK cell-mediated cytotoxicity.

Several studies have shown an association of met/val dimorphism with various diseases which include inflammatory bowel disease (24), nasopharyngeal cancer (25), and latent autoimmune diabetes (26). Table 1 summarizes all such studies in various pathological conditions involving different ethnic groups as per literature reports. Although only a limited literature is available on the role of met/val dimorphism in transplantation settings, a study by Boukouaci et al. (27) reported a strong association of MICA *val/val* genotype with increased risk of chronic graft-versus-host disease development in patients undergoing hematopoietic stem cell transplantation (HSCT). Furthermore, the same study revealed that the serum levels of soluble MICA isoform and the presence of antibodies to MICA were associated with cGvHD, which is a major complication following HSCT (27). Recently, Isernhagen et al., in a cohort of 452 patients who underwent HSCT, showed that MICA-129 met tends to increase the risk of acute GVHD (aGVHD). Presence of even one MICA-129 met allele reduced the probability of developing severe or fatal aGVHD (22). The increased risk of aGVHD was explained on the fact that the MICA-129 met variant leads to faster and more robust NKG2D signaling while the rapid downregulation of NKG2D on alloreactive CD8+ T cells explains the reduced severity of aGVHD. This effect was even more evident in patients carrying homozygous MICA-129 met alleles receiving ATG. In addition, a higher relapse rate was observed in patients with MICA-129 met as compared to those with MICA-129 val/val genotype because of reduced graft versus leukemia effect of NK and CD8+ cells consequent to downregulation of NKG2D by MICA-129 met variants. As a corollary to this, it is reasonable to hypothesize that the inflammatory processes-related abovementioned MICA features might also influence complications that occur during renal allograft rejection. Although immunologically MICA-129 dimorphism has the potential to affect graft outcome following solid organ transplantation, unlike HSCT, there is no published literature highlighting its role for the same. This certainly opens up a new area of research in renal allograft outcome.

**Table 1:** **Summary of MICA-129 dimorphism studies reported to be associated with various disease conditions in different ethnic groups**.

MICA-129 dimorphism	Year	No. of patients	Ethnicity	Disease	Association	Reference
Met/met	2005	129	Algerian	Juvenile ankylosing spondylitis	Positive	Amroun et al. (28)
Val/val	2009	130	Tunisian	Nasopharyngeal carcinoma	Positive	Douik et al. (25)
Val/val	2009	211	French	Chronic GVHD	Positive	Boukouaci et al. (27)
Met/met	2010	88	Spanish	Ulcerative colitis	Positive	Lopez-HernÂndez et al. (24)
Val/val	2011	272	Chinese	Ulcerative colitis	Positive	Zhao et al. (29)
Met/met	2011	716	Japanese	Systemic lupus erythematosus	Positive	Yoshida et al. (30)
Val/val	2012	73	Algerian	Type1 diabetes	Positive	Raache et al. (26)
Met/met	2013	340	Canadian	Cutaneous Psoriasis	Positive	Pollock et al. (31)
Met/met	2013	552	Vietanamese	Hepatocellular carcinoma	Positive	Tong et al. (32)
Met/met	2015	189	Brazil	Severity of chronic chagas disease	Positive	Ayo et al. (33)
Met/met	2015	452	Germany	Acute GVHD	Positive	Isernhagen et al. (22)

## Immune Response to MICA

The first indication that MICA could act as a new polymorphic alloantigen was provided by Zwirner et al. (34) who reported the presence of anti MICA antibodies in the sera of solid organ transplant recipients. Later, similar antibodies were reported in mice immunized with recombinant MICA (4). These investigators also demonstrated MICA as a target for complement-dependent cytotoxicity. Few years later, the landmark study by Zhang and Stastny (35) demonstrated that immunization of mice with recombinant MICA*001 having all the three extracellular domains, could elicit responses in both T and B cells. While the former showed the predominance of CD4+ T-cells, proliferating CD8+ T cells were also present and the stimulated CD8+ T cells were able to kill target cells pulsed with MICA by cell-mediated cytotoxicity. Furthermore, MICA stimulated CD4+ T cells were Th2 skewed, secreting high levels of IL-4 and correspondingly low levels of INF-γ. Thus these cells seem to provide a powerful aid to responding B cells.

Although MHC class II or class I antibodies are able to inhibit the proliferation of CD4+ and CD8+ T cells, respectively, the same is not the case with blocking of the NKG2D receptor. This led to the conclusion that T-cell response induced by MICA is confined to classical MHC molecules, which is in accordance with the indirect allorecognition of MICA peptides presented by host MHC antigens. In order to explain an efficient immune response elicited by MICA antigens despite the restricted polymorphism and much less amount of MICA on the cell surface as compared to HLA, Stastny and his group (36) proposed that “in addition to the adaptive immune response of T and B cells against an alloantigen, MICA also is capable of setting in motion the mechanisms of innate immunity. Co-stimulation by engagement of NK cells might have the effect of potentiating the T and B cell response. Another possibility might be that MICA is in itself rather immunogenic and capable of eliciting a response from a large repertoire of cells through any of a variety of mechanisms. This could result from cross-reactivity with unidentified microorganisms, expansion of the repertoire of responding immune cells, other genetic factors that might determine the magnitude of the specific immune response, or perhaps structural features of the MICA molecules that make them immunogenic.” Taken together, these findings support the concept that MICA antigens play a role in human allograft rejection by activating both humoral as well as cellular mechanisms. Furthermore, upregulation of NKG2D by interleukins, NK cell activation (in case of inflammatory conditions), NK cell-induced dendritic cell maturation, and subsequent activation of alloreactive T cells as well as NKG2D-mediated decrease in regulatory T cells could contribute to graft rejection and graft loss in transplantation (3). It may be mentioned that cellular stress-induced expression of MICA such as on renal tubules could either augment NKG2D-mediated co-stimulation of cytotoxic T cells or direct activation of alloreactive CD8+ T cells through TCR independent mechanism (3, 37). At the same time, an ever increasing amount of data has highlighted a possible association between anti-MICA antibodies and graft rejection (38). The possible mechanisms for MIC-mediated allograft rejection include development of anti-MICA antibodies, recognition of MIC on allografts, and NKG2D-mediated cytotoxicity.

## MICA Antibodies: Relevance in Solid Organ Transplantation

Following the first demonstration of the expression of MICA antigens on endothelial cells (10), attention was directed toward investigating the possibility of these molecules being a target for graft destruction in solid organ transplantation. Soon, the same group of investigators showed that antibodies in patient’s serum could specifically react with different recombinant MICA molecules (34). Others also demonstrated the expression of MICA on renal and pancreatic allograft biopsies (39) and confirmed it to be a target for complement-dependent cytotoxicity using both mouse MICA monoclonal antibodies as well as human alloantibodies (4). Similarly, in a study on 139 renal allograft recipients, Sumitran-Holgersson et al. (40) showed a significant correlation of MICA antibodies with graft loss. Thus, the year 2002 was an important milestone in providing evidence that MICA expression in graft tissues could lead to antibody-mediated lysis and that MICA antibodies could have an important role in precipitating antibody-mediated rejection (AMR). This was followed by a lull period of 3 years before Mizutani and coworkers (41) published a retrospective study of “serial ten-year follow up of HLA and MICA antibody production prior to kidney graft failure” providing evidence that patients who had both antibodies rejected their grafts more frequently than those who did not have either of these antibodies. Another study by the same authors indicated that MICA antibodies detected at pretransplant period could have a role in the development of AMR (42). The above mentioned studies along with the development of more reliable and convenient Luminex bead based assay system opened the floodgates for several studies investigating the relevance and impact of MICA antibodies on allograft outcome. Hence, the year 2007 witnessed a surge in studies investigating the effect of MICA antibodies on graft outcome in solid organ transplants, more so in kidney and heart transplants. Table 2 summarizes relevant studies on the influence of MICA antibodies on graft outcome in various solid organ transplantations.

**Table 2:** **Presence of MICA antibodies and their effect on allograft outcome in solid organ transplantation**.

Organ	Detection time	Year	Number of patients	Transplant (DD/LD)	Follow-up (duration)	Reference	Outcome
Kidney	Pre-tx	2002	139	DD	3 months	Sumitran-Holgersson et al. (40)	↑AMR
		2007	1,910	DD	1 year	Zou et al. (43)	↑AMR, ↓GS
		2010	425	NS	1, 5, and 10 years	Lemy et al. (44)	↔
		2012	40	LD	1 year	Solgi et al. (45)	↔
		2013	727	DD + LD	3, 6, 12, and 24 months	Sánchez-Zapardiel et al. (46)	↑AMR
	Post-tx	2005	145	DD + LD	10 years	Mizutani et al. (41)	↓GS
		2007	185	LD		Panigrahi et al. (47)	↑AMR
		2007	1,921	DD + LD	4 years	Terasaki et al. (48)	↓GS
		2009	284	DD	3 years	Suarez-Alvarez et al. (49)	↑AMR
		2011	442	DD + LD	5.9 years (mean)	Cox et al. (50)	↑CR
		2012	779	DD + LD	4 years	Lemy et al. (51)	↔
		2012	147	DD + LD	6 months	Seyhun et al. (52)	↔
Heart	Pre and post-tx	2007	44	DD	1 year	Suarez-Alvarez et al. (53)	↑AMR
	Pre-tx	2009	491	DD	1 and 5 years	Smith et al. (54)	↔AMR/CAV ↑GS
	Pre-tx	2010	63	DD	6 months	Pavlova et al. (55)	↔
	Post-tx	2010	95	DD	1.8 and 8.9 years (mean)	Nath et al. (56)	↑AMR, ↑CAV
	Post-tx	2011	168	DD	2 years (median)	Zhang et al. (57)	↑AMR
		2015	05	Animal experiments (rat-to-mouse cardiac transplantation model)		Yu et al. (58)	↑AR
Liver		2008	84	NS	2 years	Uzunel et al. (59)	↔
		2013	123	NS	7 years	Ciszek et al. (60)	↔

*MICA, major histocompatibility complex class I chain-related molecule A; AT1R, angiotensin II type 1 receptor; AECA, anti-endothelial cell antibody; Col V, collagen V; KA1T, k-α1 tubulin; LSECs, liver sinusoidal endothelial cells; AMR, antibody-mediated rejection; GS, graft survival; AVR, acute vascular rejection; CAV, cardiac allograft vasculopathy; BOS, bronchiolitis obliterans syndrome; DD, deceased donor; LD, live donor; NS, not specified; AR, acute rejection; ↔, no adverse effect*.

## Renal Transplantation

### Impact of Pretransplant MICA Antibodies

Exact mechanisms by which individuals develop antibodies to MICA are largely unknown. Although, pregnancy *per se* and previous transplants can sensitize the patient leading to the development of anti-MICA antibodies (34), the role of blood transfusions in their induction is not fully clear (43, 44).

The first major study to evaluate the potential association of MICA antibodies with overall allograft survival was conducted by Zou et al. (43). It was an international collaborative study involving 20 centers in 13 countries with pre transplant serum samples obtained from 1,910 patients. The experiment was performed blindly by testing MICA antibodies without any knowledge of the clinical course. The results showed that at least 217 of the 1,910 patients (11.4%) had MICA antibodies and their 1-year graft survival (GS) was 88.3% as compared to 93% in the group without MICA antibodies (*p* = 0.01). Among patients of primary renal grafts, survival was even lower (87.8%) compared with 93.5% for those who did not have MICA antibodies (*p* = 0.005). Interestingly the association of MICA sensitization with GS was observed in patients well matched for HLA. Independent analysis of 326 patients with 0 or 1 HLA-A, -B, or DR mismatches also showed that recipients with MICA antibodies had poorer GS of 83.2% compared to 95.1% of those without MICA antibodies. However, the study did not investigate the impact of possible confounding factors that are likely to influence graft loss.

Subsequently, Lemy and colleagues (44) studied for the presence of MICA antibody in sera from 494 healthy controls and 597 patients with chronic kidney disease (CKD) stage V and reported threefold higher prevalence of MICA antibodies in patients with CKD when compared with controls. Using logistic regression analysis involving subsets of patients free of transfusions and transplantation also revealed at least twofold higher prevalence of MICA antibodies in CKD patients when compared to healthy controls. It is intriguing that these antibodies were more frequent in males rather than females in the cohort as a whole (14 versus 7%) and also among individual groups despite pregnancy being an independent risk factor for their development. Thus factors remaining significantly associated with MICA antibodies after logistic regression analysis were blood transfusions, previous transplantation, and females with two or more pregnancies. The finding of blood transfusion as a significant sensitizing event was in sharp contrast with the findings of Zou although five transfusions were required for categorization as “transfused” compared to only one in Zou’s study (43). Another very interesting finding of this study was that no sensitizing events could be identified in a third of the patients with MICA antibodies and CKD stage V, implicating other possible mechanisms for MICA sensitization. Additionally, 20% of CKD patients had MICA antibodies that were auto-reactive, a rare finding with HLA antibodies (61).

It is important here to debate on the results of two important studies—one carried out by Zou et al. (43) and the other by Lemy and colleagues (44). Even though the broad design of the two studies has been similar with pretransplant testing for MICA antibodies, the outcomes were dissimilar in terms of GS. Furthermore, there were differences in the number of patients included in the two studies, but the latter group of investigators found better survival in patients positive for MICA antibodies, albeit insignificantly. However, an analysis of immunosuppression protocols between the two studies showed that Lemy’s group of patients were more heavily immunosuppressed and that could make an effect on the incidence and impact of MICA antibodies. Others also failed to show significantly higher rejection rates in patients expressing MICA antibodies as compared to those who did not express (62). Similarly, Solgi et al. (45) did not find significant difference in rejection episodes on comparing patients with or without the presence of anti-MICA antibodies. A retrospective study involving 727 renal allograft recipients published by Sánchez-Zapardiel et al. (46) revealed a 7.15% prevalence of MICA antibodies in patients waiting for a renal transplant. They reported that preformed anti-MICA antibodies significantly increased the risk for allograft rejection particularly early after transplantation and that this effect was independent of the presence of anti-HLA antibodies. However, no significant difference was noticed in allograft survival and rejection rates at 2-year follow-up. Moreover, no significant epidemiological or clinical differences were observed between MICA antibody positive and negative groups. The study did not define the donor specificity of anti-MICA antibodies. The same group of authors further demonstrated that presence of anti-MICA antibodies at pretransplant periods can bind native MICA molecules on the cell membrane and was able to mediate cell death by fixing and activating the complement cascade by using both the C1q single-antigen beads assay and complement-dependent cytotoxicity (63).

Our experience with live donor renal transplantation (64) is very similar to that of others (65, 66) suggesting that presence of pretransplant MICA antibodies especially those against donor antigens with MFI in the range of 10,000–20,000 are capable of causing hyperacute and acute rejection (AR). Clearly, there are gap areas and lack of consensus on the epidemiology and specificity of MICA antibodies on the one hand and their impact on AR and GS on the other.

### Impact of Posttransplant MICA Antibodies

The issue of *de novo* occurrence of MICA antibodies posttransplantation has been a subject of intense investigation. In a study involving 185 consecutive live related donor renal transplant patients, we analyzed posttransplant serum samples at varying time periods and reported a significant decline in 2-year GS if both HLA and MICA antibodies were detected (47). The survival was only 17% compared to 89% of those with no antibodies. Furthermore, patients with either MICA or HLA antibodies alone also had a significantly reduced GS of 71% as compared to the no antibody group.

Simultaneously, a large collaborative international study coordinated by Paul Terasaki tested sera for both HLA and MICA antibody production from 1,329 recipients of renal grafts (964 from deceased donors and 365 living donors) from 21 participating centers as a part of the 13th International Histocompatibility and Immunogenetics Workshop Conference (IHIWC) and 22 centers as a part of the 14th IHIWC (48). Only those recipients who did not produce HLA antibodies pretransplant (pretransplant testing for MICA antibodies was not performed) and who survived for more than 6 months were included in this study. HLA antibodies were detected with CDC, ELISA, or Luminex techniques, while MICA antibodies were detected using eight different recombinant MICA molecules produced in HMY2.C1R cells, isolated and coated on Luminex beads. In the 13th workshop, the 4-year deceased donor GS among 806 patients who were negative for HLA antibodies was 81% as compared to 58% for 158 recipients with HLA antibodies (*p* < 0.0001) and 72% for 69 patients with the presence of MICA antibodies (*p* = 0.02). Among those with living donor grafts, 4-year GS was 78% for 275 patients without HLA antibodies, 62% for 90 patients with HLA antibodies (*p* = 0.0008), and 80% for 21 patients with MICA antibodies (*p* = NS). In the 14th workshop, 1-year GS for deceased donor recipients without MICA antibodies was 96.8% as compared to 82.7% for 33 patients with MICA antibodies alone (*p* = 0.0005). However, the same was not observed with living donor recipients as 19 patients with MICA antibodies had 100% 1-year GS. Multivariate analysis at both time points revealed that MICA antibodies were significantly and independently associated with reduced GS in deceased donor grafts, providing strong evidence for the involvement of these antibodies with graft rejection. It may be mentioned that these studies did not consider previous AR episodes or other confounding factors that are also likely to influence graft loss. The explanation for the lack of significance of MICA antibodies in living donor transplants was attributed to the limited number of patients in the study group.

Up until around 2009, the specificity of MICA antibodies and epitopes recognized by them had received very little attention. Gautier et al. (67) performed MICA typing of 43 recipient–donor pairs of patients undergoing third renal transplant and also evaluated MICA antibody using the LABScreen SAB Luminex method (One Lambda). The antibody screening was done on the day of transplant and after 1 year. They observed greater frequency of patients with two MICA mismatches among those who developed rejection; whereas all patients with graft losses had 0 or 1 MICA mismatch. Antibodies specific to donor MICA antigens (MICA-DSA) were found to be equally associated with functional and failed grafts. In this study, although MICA genotyping was attempted on all patients and donors including those positive for MICA antibodies, the authors did not examine the nature of mismatches between those who produced *de novo* MICA antibodies and those that did not.

At around the same time, Suarez-Alvarez and colleagues (49) combined a clinical study of MICA antibody production in deceased donor renal transplantation along with MICA epitope analysis. Posttransplant sera of 284 patients were tested for MICA antibodies using Luminex technology, and patients were followed for up to 3 years. The results revealed presence of MICA antibodies alone without the presence of anti-HLA antibodies in 30 (10.6%) patients. Furthermore, 29.6% of patients who developed AR had MICA antibodies as compared to 13.3% of the antibody negative group (*p* < 0.05). Using epitope mapping with a synthesized library of overlapping peptides from the extracellular domains of MICA molecules, the investigators determined nine antigenic regions reactive with MICA antibodies in patient’s serum. Four of these regions were mapped to variable sites in the molecule with polymorphic amino acids while five antigenic regions located in constant region had shared epitopes found in all MICA alleles.

Others used a novel technique to detect *de novo* HLA and MICA antibodies in 15 patients following renal transplantation (68). Pre- and posttransplant sera were profiled using the Invitrogen Protoarray Human Protein Microarray platform containing 5,056 non-redundant human proteins, purified from insect cells. For the purpose of analysis, three main clinical phenotypes with five patients each were considered (i) the first group comprised of patients positive for C4d and undergoing AR, (ii) the second group with cellular rejection were negative for both donor-specific antibodies (DSA) and C4d, and (iii) the third group consisted of patients with stable graft function without any rejection episodes. The results revealed *de novo* occurrence of MICA antibodies in 11 of the 15 patients with the mean antibody signal intensity being higher in those with C4d+ AR as compared to those with C4d− AR. Additionally, integrative genomics predicted localization of MICA antigen to the glomerulus in the normal kidney. Immunohistochemistry confirmed the finding that MICA antigens preferentially localized to glomerular podocytes. MICA expression in normal kidney podocytes may actually be a means to resist NK cell-mediated cytotoxicity. These investigators also showed the induced expression of MICA *in vivo* on infiltrating lymphocytes during rejection episodes suggesting that MICA antibody-mediated immune responses occurred irrespective of graft rejection and that antibody levels increase during AMR but not cellular rejection. Therefore, keeping in view the significant rise in antibody titers prior to and during humoral rejection, serial measurement of MICA antibody levels rather than checking cross-sectionally at the time of rejection may be more useful. Besides the observed correlation between C4d+ AR and high MICA levels, the latter were also significantly associated with MHC class II-specific circulating DSA. Since an association of HLA-DSA class II with development of chronic glomerular injury is already established, it is possible that anti-MICA antibodies may be playing a potentiating role in the pathogenesis of chronic transplant glomerulopathy.

Whether there is an influence of MICA allele mismatching on antibody production and graft rejection is not fully clear? Cox and coworkers (50) screened 442 renal transplant recipients for MICA antibodies using three different Luminex-based single antigen kits—One Lambda, Gen-Probe, and an “in-house” assay. Mean time for testing of antibodies was 7 months after transplantation and the mean follow-up period was 5.9 years. MICA antibody specificities were considered positive only if confirmed by at least two different kits. In 227 of the above recipient-donor pairs, MICA allele typing was performed by DNA sequencing. At least 17 recipients (7.5%) developed MICA antibodies of which 10 had *de novo* DSA. Moreover, eight of these MICA+ recipients and seven of those who had *de novo* DSA had no HLA antibodies. On multivariate analysis, MICA mismatching was found to be an independent significant factor associated with the development of MICA antibodies. Also the presence of both MICA and HLA antibodies together significantly correlated with ACR but not AMR, although occurrence of MICA antibodies alone failed to show an association with either of these events. Nevertheless, recipients with MICA DSA alone showed a significant association with graft dysfunction (↓ eGFR) 2 years following transplantation, as were those with HLA-DSA alone who showed significantly reduced eGFR after 3 years. Thus the kinetics of antibody response in this study pointed toward an accelerated graft dysfunction in the presence of MICA antibodies.

In a retrospective study performed by Lemy et al. (51) on 1-year posttransplant sera from 779 renal transplant recipients, a 5.4% prevalence of MICA antibodies was observed. MICA+ patients were more frequently HLA sensitized and had to undergo re-engraftment. There was no significant difference in 4-year death-censored GS between MICA positive and negative patients (97 versus 94%, *p* = 0.28). By Cox multivariate analysis, graft loss was found to be independently associated with the number of HLA-DR mismatches, AR within the first year posttransplantation, 1-year serum creatinine ≥1.5 mg/dl, and the presence of HLA antibodies at 1 year, but not the presence of MICA antibodies. Another study comprising of 84 renal allograft recipients with a follow-up of 4 years reported that more than one-third of the recipients developed antibodies to HLA and/or MICA and the percentage of recipients who developed *de novo* antibodies increased with time after transplantation elapsed. Recipients positive for these antibodies had higher serum creatinine levels and worse allograft function than those without antibodies (69).

## Heart Transplantation

A number of studies have shed light on the correlation of MICA antibodies to cardiac allograft rejection episodes. Suarez-Alvarez (70) demonstrated significant correlation between the presence of anti-MICA antibodies detected by CDC using recombinant cell lines and AR following heart transplantation. A year later, the same group performed another study to investigate a possible relationship between MICA antibody production and heart allograft rejection in 44 recipients (53). This time, MICA antibodies were detected using both MICA transfected cell lines in a CDC assay and a commercial assay using Luminex beads. While a quarter of the patients were antibody positive by the CDC technique, only seven (15.9%) showed MICA antibody by the Luminex assay. Nine patients had rejection and a majority of them (60%) were positive for MICA antibodies by the CDC method as compared to five patients (14.3%) without rejection (*p* = 0.0038). Analysis by Luminex revealed 55.5% of AR patients as compared to only 6% without rejection had MICA antibodies (*p* = 0.0020). They also performed MICA allele DNA-typing for donors and recipients where the recipient was positive for MICA antibodies. All patients with MICA antibodies and AR had MICA-DSA although five patients also had autoantibodies. This study was limited by the small number of patients; nevertheless it was the first study to show a possible correlation between MICA-DSA and AR. Additionally, they determined MICA mRNA levels in endomyocardial biopsies obtained from 10 cardiac transplant recipients and found these to be higher in biopsies showing rejection than those without it. In majority of the cases, MICA expression was higher immediately following transplantation, independent of the rejection event, suggesting an upregulated antigen expression due possibly to cellular stress.

In contrast to the above, Smith and coworkers (54) in their study on 491 heart transplant recipients did not find any significant correlation of pre- or posttransplant MICA antibodies or MICA-DSA with cardiac allograft survival, AR episodes, or cardiac allograft vasculopathy (CAV). Similar observations were made by Pavlova et al. (55) who reported from their study of 68 heart transplant recipients that patients with pretransplant MICA antibodies did not significantly associate with AMR or ACR, although a trend was observed with AMR (*p* = 0.06). Others however demonstrated a significant association of anti-MICA antibodies with AR and CAV (56). These investigators also showed that development of HLA-DSA preceded the detection of MICA antibodies. An apparent explanation given by the authors was that this could be because of binding of HLA-DSA to the allograft giving rise to inflammatory cascade, which may result in upregulation of MICA antigens, alloreactivity, and sensitization. Another study found a significant correlation between the presence of MICA-DSA with AMR, while anti-MICA antibodies that were not donor specific (NDSA) did not correlate (57). In this study, 10% of the patients developed autoantibodies to MICA, but these did not associate with the development of AMR. Using an allogeneic animal model system involving rat-to-mouse cardiac transplants, Yu and coworkers (58) reported high MICA expression in recipients’ heart and provided evidence to show that anti-MICA antibodies in their sera were associated with high risk of AR.

## Liver Transplantation

There are only limited studies defining the role of MICA antibodies in liver transplantation. A study of MICA antibody production in liver allograft recipients did not reveal an association with allograft rejection (59). Histological analysis revealed that MICA is not normally expressed on liver cells and its expression is not induced during rejection episodes. Ciszek and coworkers (60) analyzed the impact of anti-HLA and anti-MICA antibodies in 123 ABO compatible liver transplant recipients with a follow-up of 7 years. They reported that neither the presence of anti-HLA nor anti-MICA antibodies correlated with acute graft rejection or GS.

## Soluble MICA (sMICA): Role in Solid Organ Transplantation

**Figure 3:**
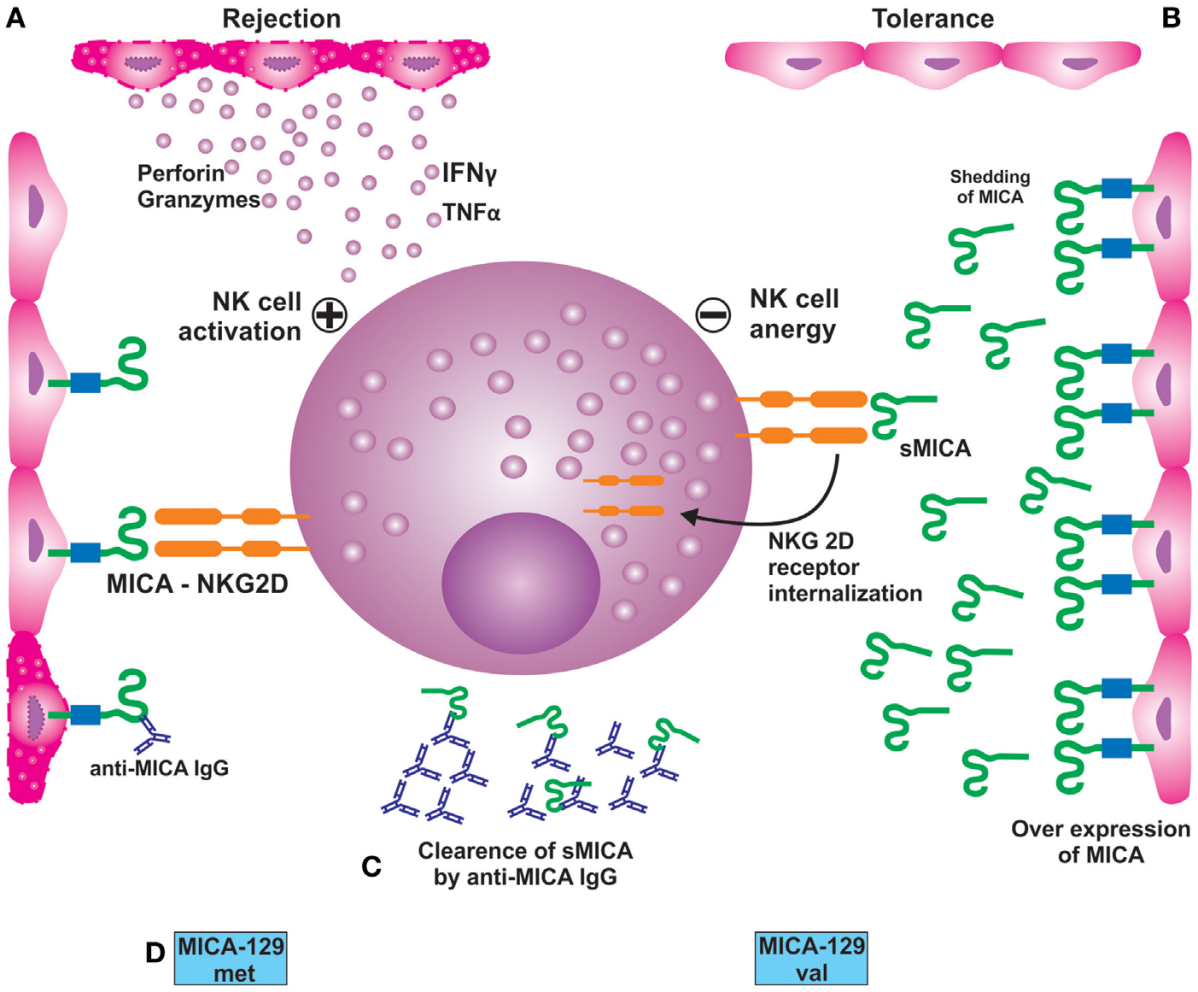
**(A)** Interaction of NKG2D receptor with its ligand MICA, expressed on endothelial cells, results in activation of NK cells leading to their damage. **(B)** Over expression of MICA on endothelial cells membrane and its proteolytic shedding from the surface leading to the formation of sMICA. The latter interacts with the NKG2D receptor, resulting in its internalization and subsequent suppression or impairment of NKG2D-mediated immune response; **(C)** preformed or *de novo*-developed MICA antibodies recognize MICA antigens present on the allograft leading to acute or chronic rejection; **(D)** potential impact of MICA-129 met/val dimorphism on renal graft outcome. Patients expressing met/met in homozygous state are prone to rejection episodes than those with val/val genotype.

In addition to the membrane bound form, a soluble isoform of MICA (sMICA) derived from the proteolytic shedding of membrane bound molecule appears in the serum. MICA, a ligand for NKG2D receptors, forms a complex with ERp5, a disulfide isomerase/chaperone and induces a conformational change enabling proteolytic cleavage of MICA by ADAM proteases. The interaction of NKG2D by the sMICA results in the endocytosis and degradation of receptor–ligand complex and thus suppresses NKG2D-mediated host innate immunity (Figure 3).

Most studies on soluble MICA release in the serum have been directed toward understanding their influence on tumor growth, with very little literature available on the associated biology. The intricate nexus between the science behind sMICA role in cancers and transplant rejection has been highlighted through a few studies. For example, Suarez-Alvarez et al. (70), while evaluating the role of MICA on heart graft acceptance, demonstrated an inverse relationship between sMICA levels and AR. The study was conducted on 31 heart transplant recipients with a follow-up of 1 year, of which 8 patients suffered AR while the remaining 23 patients did not develop AR. Further analysis showed that 17 out of 23 patients without AR had detectable levels of sMICA as compared to two patients in the rejected group (*p* < 0.03). On combined analysis of MICA antibodies and sMICA, the authors found tendency for MICA antibodies to occur in the absence of sMICA in the AR group of patients. Conversely, the sMICA levels were detected in patients without MICA antibodies and in absence of AR. These authors published another paper in the same year, where they monitored sMICA levels in pretransplant serum samples and at 15 days, 3 months and 1 year posttransplantation, in 34 heart transplant recipients (71). sMICA was practically absent in the pretransplant sera, while it was detected in 21 patients at 15 days posttransplantation. Interestingly, 20 of these 21 patients did not develop AR (*p* = 0.0001), whereas 9 of the 13 patients, in whose serum sMICA was not detected, developed AR. These observations are in conformity with the previous study suggesting that presence of sMICA contributes to better graft acceptance. Recent experiment conducted on an animal model (rat-to-mouse cardiac transplantation) also demonstrated a negative association of sMICA with AR. The investigators reported that xenografts having anti-MICA antibodies and experiencing AR tended to develop in the absence of sMICA (58). Assadiasl and coworkers (72) in their study on 30 each patients of coronary artery disease and transplant recipients with stable grafts and 15 healthy controls did not find any significant difference in the presence and amount of soluble MICA between the three groups.

Zou et al. (73), in an attempt to assess the effect of sMICA on the outcome of liver transplantation, evaluated levels in pre- and posttransplant sera from 133 consecutive primary liver transplant patients and in sera from 88 healthy volunteers using sandwich ELISA. The study revealed that 37.6% of recipients had significantly higher pretransplant sMICA than the healthy population, while recipients with decreased posttransplant sMICA following liver transplantation had a lower incidence rate of biliary cast syndrome (BCS) than those with sustained high levels of sMICA after transplantation (10.5 versus 38.7%, *p* = 0.0302) suggesting that dynamic changes in these levels are associated with BCS development.

Clearly, there is a general dearth of published literature evaluating a possible correlation between circulating levels of sMICA and graft outcome in solid organ transplantation. Studies involving larger cohorts and diverse ethnic groups are needed to determine the applicability of sMICA as a potential biomarker of prognostic importance in solid organ transplantation.

## Conclusion

Despite clear indications of MICA antibodies impacting graft outcome adversely, a definitive consensus on this relationship is yet to be arrived. Furthermore, only a few studies have dealt with the impact of MICA-DSA as compared to those that are NDSA on graft outcome. Two factors are important while analyzing the role of MICA antibodies: (i) currently employed pretransplant crossmatch procedures are not sensitive enough to detect MICA DSA and (ii) the currently used immunosuppressants for induction and maintenance may not be effective in suppressing the immune response against MICA antigens because they are all directed at suppression of T cell response albeit through different mechanisms. Data so far suggest that circulating levels of soluble MICA could well prove to be a potential biomarker of prognostic importance in the assessment of patients after renal transplantation. At the present moment, there is scarcity of published literature evaluating a possible correlation between production of sMICA and their titers with graft outcome in renal transplantation. Further studies involving larger cohorts and diverse ethnic groups could help to reinforce the current findings. Our data on MICA-129 dimorphism adds another dimension in defining its exact role and influence following solid organ transplantation.

## Author Contributions

AKB and NKM designed and wrote the paper. NKM provided excellent inputs and advice.

## Conflict of Interest Statement

The authors declare that the review article has been prepared independent of any commercial or financial relationships and without any potential conflict of interest.

## Footnotes

^1^http://hla.alleles.org/nomenclature/stats.html, April 14, 2016.

^2^http://hla.alleles.org/nomenclature/stats.html, April 14, 2016.
